# Ca^2+^ increases cardiac muscle viscoelasticity independent of active force development

**DOI:** 10.1016/j.bpj.2025.07.009

**Published:** 2025-07-12

**Authors:** Anthony J. Baker, On Yeung Li, Filip Ježek, Paul C. Simpson, Naomi C. Chesler, Daniel A. Beard

**Affiliations:** 1 Veterans Affairs Medical Center, San Francisco, California; 2 Department of Medicine, University of California, San Francisco, San Francisco, California; 3 Department of Molecular and Integrative Physiology, University of Michigan, Ann Arbor, Michigan; 4 Edwards Lifesciences Foundation Cardiovascular Innovation and Research Center and Department of Biomedical Engineering, University of California, Irvine, Irvine, California

## Abstract

In addition to activation of muscle contraction by Ca^2+^, previous studies suggest that Ca^2+^ also affects muscle passive mechanical properties. The goal of this study was to determine if Ca^2+^ regulates the stiffness of cardiac muscle, independent of active contraction. The mechanical response to stretch for mouse demembranated cardiac trabeculae was probed at different Ca^2+^ levels after eliminating active contraction using a combination of two myosin ATPase inhibitors: *para*-nitroblebbistatin (PNB; 50 *μ*M) plus mavacamten (Mava; 50 *μ*M). Myocardial force level was assessed during large stretches (≈20% initial muscle length) with a range of stretch velocities. For relaxed muscle, in response to stretch, muscle force rose to a peak and then decayed toward a lower steady-state level. Peak force was higher with faster stretch velocity, consistent with the presence of a viscoelastic element. However, the steady-state force was independent of stretch velocity, consistent with the presence of an elastic component. In the presence of the inhibitors PNB plus Mava, when the Ca^2+^ level was increased, active contraction was completely prevented. However, the viscoelastic force response to stretch was markedly increased by high Ca^2+^ and was >sixfold higher than at the low Ca^2+^ level. The relationship of viscoelastic force to Ca^2+^ level had a similar form to the relationship of active force to Ca^2+^ (measured in the absence of inhibitors), suggesting that a common regulatory mechanism is involved. As expected, Ca^2+^-activated contraction was inhibited by lowering the temperature from 21°C to 10°C. In contrast, the Ca^2+^-activated viscoelastic property was not inhibited at lower temperatures, further suggesting that active contraction and the viscoelastic property involve distinct mechanisms. This study demonstrates that in addition to triggering activation of contraction, Ca^2+^ also increases the apparent viscoelastic property of cardiac muscle.

## Significance

Ca^2+^ is well known to trigger activation of muscle contraction. This study demonstrates a new mechanical role for Ca^2+^ in cardiac muscle involving a >sixfold increase in the apparent muscle viscoelasticity. Activation of a viscoelastic element by Ca^2+^ might influence the mechanical properties of activated cardiac muscle.

## Introduction

Previous studies suggest that the passive mechanical properties of skeletal and cardiac muscle are modulated by interactions occurring within muscle sarcomeres between the giant elastic protein titin and actin filaments (1,2,3). Moreover, titin-actin interactions are sensitive to the level of muscle activation by Ca^2+^ (1,2,4,5,6). Recently, electrical stimulation of frog skeletal muscle was reported to increase the resistance of muscle sarcomeres to stretch, independent of active force development (7). This phenomenon was attributed to the effects of muscle activation, likely due to increased intracellular-level Ca^2+^, on the mechanical properties of titin and on titin’s interactions with other sarcomeric proteins. Given the importance of cardiac muscle passive mechanical properties in health and disease (8,9), the goal of this study was to determine if and how the passive mechanical properties of cardiac muscle are sensitive to levels of Ca^2+^. Using demembranated cardiac trabeculae from male and female mice, we measured muscle force during and after muscle stretches that were imposed with a range of velocities. To measure the effect of Ca^2+^ in the absence of force production, we prevented cross-bridge cycling using a combination of two inhibitors: *para*-nitroblebbistatin (PNB) and mavacamten (Mava) (10,11). The combination of these inhibitors prevented Ca^2+^-activated force development. As previously reported, we found that the dynamic force response of cardiac muscle to stretch consists of a velocity-sensitive viscoelastic component and a velocity-insensitive elastic component (12,13,14,15). Importantly, at the fastest stretch velocity, we found that the viscoelastic component of the force response was >sixfold higher at the high Ca^2+^ level than at the low Ca^2+^ level. In summary, for cardiac muscle, activation by Ca^2+^ caused a large increase in a muscle viscoelastic property that was independent of active force development.

## Materials and methods

The study was approved by the Animal Care and Use Subcommittee of the San Francisco Veterans Affairs Medical Center and conformed to the Guide for the Care and Use of Laboratory Animals published by the National Institutes of Health (revised 2011). This institution is accredited by the American Association for the Accreditation of Laboratory Animal Care (the Institutional PHS Assurance number is A3476-01).

### Demembranated right ventricular trabeculae

Trabeculae were prepared as we recently described (16) using 12-week-old male and female C57BL/6J mice (Jackson Labs, Bar Harbor, Maine). Briefly, hearts were removed from deeply anesthetized mice (3% isoflurane), placed in cold arrest solution, and flushed with a modified Krebs solution (16). A piece of the right ventricle near the tricuspid valve that contained a trabecula was dissected and immersed in ice-cold relaxing solution (see below) plus 2% Triton X-100 (Sigma-Aldrich, St. Louis, Missouri) for 1 h, washed in ice-cold relaxing solution for 1 h, and then stored at −20**°**C for up to 85 days (mean: 24 days) in a 1:1 mixture of relaxing solution and glycerol (17,18).

#### Solutions

Ca^2+^-free relaxing solution (denoted pCa 11) contained (in mM) 20 EGTA, 8 MgATP, 12 creatine phosphate, and 100 N,N-bis[2-hydroxyethyl]2-aminoethane sulfonic acid; pH adjusted to 7.1 with KOH, ionic strength adjusted to 200 mM with KCl, and a temperature of 21°C (19). Preactivating solution was identical but with calcium buffering reduced by replacing 19.5 mM EGTA with hexamethylenediamine-N,N,NV,NV-tetraacetate (Fluka, Seelze, Germany). Activating solution (pCa 4.51) contained 20 mM Ca^2+^EGTA. Relaxing and activating solutions were mixed to obtain solutions with intermediate pCa (20). All solutions contained 1% (v/v) protease inhibitor cocktail P-8340 and 10 IU/mL creatine kinase (Sigma, St. Louis, Missouri).

To inhibit myosin cross-bridges, a combination of 50 *μ*M PNB plus 50 *μ*M Mava was added from 10 mM stock solutions dissolved in DMSO (1% DMSO final).

#### Myofilament extraction

Treatment of cardiac trabeculae with a high-salt extraction protocol removes >95% of myosin and actin, which anchor titin in the sarcomere (21). After extraction, the mechanical properties of muscle should be determined primarily by non-myofilament structures, especially collagen. After mechanical studies, trabeculae were incubated with relaxing solution containing 0.6 M KCl for 1 h, followed by relaxing solution containing 1 M KI for 1 h at 21°C (21). Muscles were washed in relaxing solution for 60 s, and mechanical tests were repeated.

#### Mechanical studies

A demembranated trabecula (one per mouse) was attached using aluminum t-clips to a force transducer (Model 400, Aurora Scientific, Ontario, Canada) and a computer-controlled servo-motor in a small glass-bottomed chamber of a permeabilized fiber test system (model 1400A, Aurora Scientific) on an inverted microscope with a video system (model 900B, Aurora Scientific) to measure the sarcomere length using a 40× objective. The temperature was set to 21°C for mechanical studies. The effect of lowered temperature (10°C) was also determined.

In the relaxing solution, the initial muscle length (Lo) was adjusted to set the sarcomere length to 2.0 *μ*m. Trabecula dimensions were measured and used to normalize muscle force to the muscle cross-sectional area (assuming an elliptical cross-section). Then, maximal Ca^2+^-activated force (Fmax) was measured by moving the trabecula to preactivating solution for 60 s, then activating solution for 6 s, and finally returned to the relaxing solution.

In relaxing, activating, and intermediate pCa solutions, and in the presence of 50 *μ*M PNB plus 50 *μ*M Mava, trabeculae were subjected to constant velocity stretches from 0.95 to 1.175 Lo with stretch durations of 100, 10, 1, and 0.1 s. After each stretch, muscle length was held constant for 60 s and then briefly reduced to 0.8 Lo to slacken the muscle and establish the zero force level. Between stretches, muscles were equilibrated for 60 s at a length of 0.95 Lo. Such large-magnitude stretches have been commonly used to probe the diastolic properties of cardiac muscle samples (21,22,23).

The relationship between muscle viscoelastic force (Fη) and stretch velocity (V) was fit to the dose-response relation: Fη = Fη_min_ + V × (Fη_max_ − Fη_min_)/(V_50_ + V), where Fη_min_ is the minimum value of Fη (in relaxing solution) and V_50_ is the velocity that results in a half maximal increase of Fη.

For measurements obtained at the fastest stretch velocity (2.25 muscle lengths per s [ML/s], stretch duration 0.1 s), the relationship between muscle viscoelastic force (F^∗^η) and [Ca^2+^] was fit to the Hill equation: F^∗^η = F^∗^η_max_ × [Ca^2+^]^*n*H^/([Ca^2+^]^*n*H^ + EC_50_^*n*H^), where F^∗^η_max_ is the maximum Ca^2+^-activated viscoelastic force, EC_50_ is the [Ca^2+^] at which F^∗^η is 50% of F^∗^η_max_, and *n*H is the Hill coefficient, reflecting the slope of the relationship at EC_50_.

Finally, in a subset of two experiments, the relationship between the developed force and [Ca^2+^] was compared to that of F^∗^η versus [Ca^2+^]. Developed force in the absence of inhibitors was measured at the same muscle length (1.175 Lo) used to measure F^∗^η.

### Sex differences

We compared stiffness properties of right ventricular trabeculae from 10-week-old adult male versus female mice. For males, body weight was greater (23.9 ± 0.6 g, *n* = 3) than for females (20.6 ± 0.6 g, *n* = 3, *p* = 0.017). Trabecula length (783 ± 72 *μ*m, *n* = 6) and width (154 ± 24 *μ*m, *n* = 6) were similar in males and females. There was a trend for muscle thickness to be greater in males (108 ± 7 *μ*m, n = 3 *μ*m) than females (87 ± 4 *μ*m, *n* = 3, *p* = 0.056).

### Statistical analysis

Data are presented as mean ± SE. Statistical tests (one-way and two-way ANOVA, paired and unpaired *t*-tests) were performed using Prism 10 software (GraphPad Software, La Jolla, California) with a significance level set at *p* < 0.05.

## Results

### Viscoelastic force response to stretch

**Figure 1:**
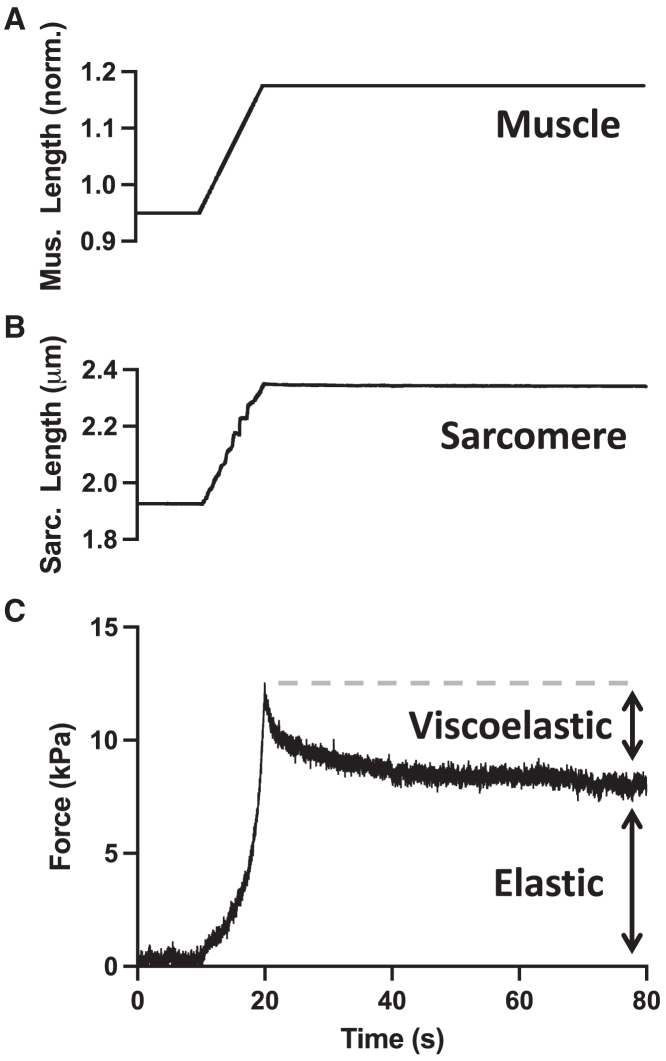
Viscoelastic property of cardiac muscle. Example records of changes in muscle length (*A*), sarcomere length (*B*), and muscle force (*C*) in response to a linear muscle stretch under relaxing conditions. The response to stretch had viscoelastic and elastic components. Muscle length was normalized to a length at which the sarcomere length was 2.0 *μ*m.

Fig. 1 shows typical records of muscle length, sarcomere length, and muscle force from a relaxed trabecula subjected to a large ramp stretch of 10 s duration. Muscle length was linearly increased from 0.95 to 1.175 Lo, associated with an increase of sarcomere length from ≈1.9 to 2.3 *μ*m. In line with previous reports, stretch caused force to increase to a peak and then relax to a quasi-steady-state level after the stretch (12,13,14,15). The viscoelastic force response to stretch was quantitated from the difference between the peak force at the end of the stretch and the quasi-steady-state level of force reached 60 s after the stretch. This stress relaxation did not involve an appreciable change in sarcomere length.

**Figure 2:**
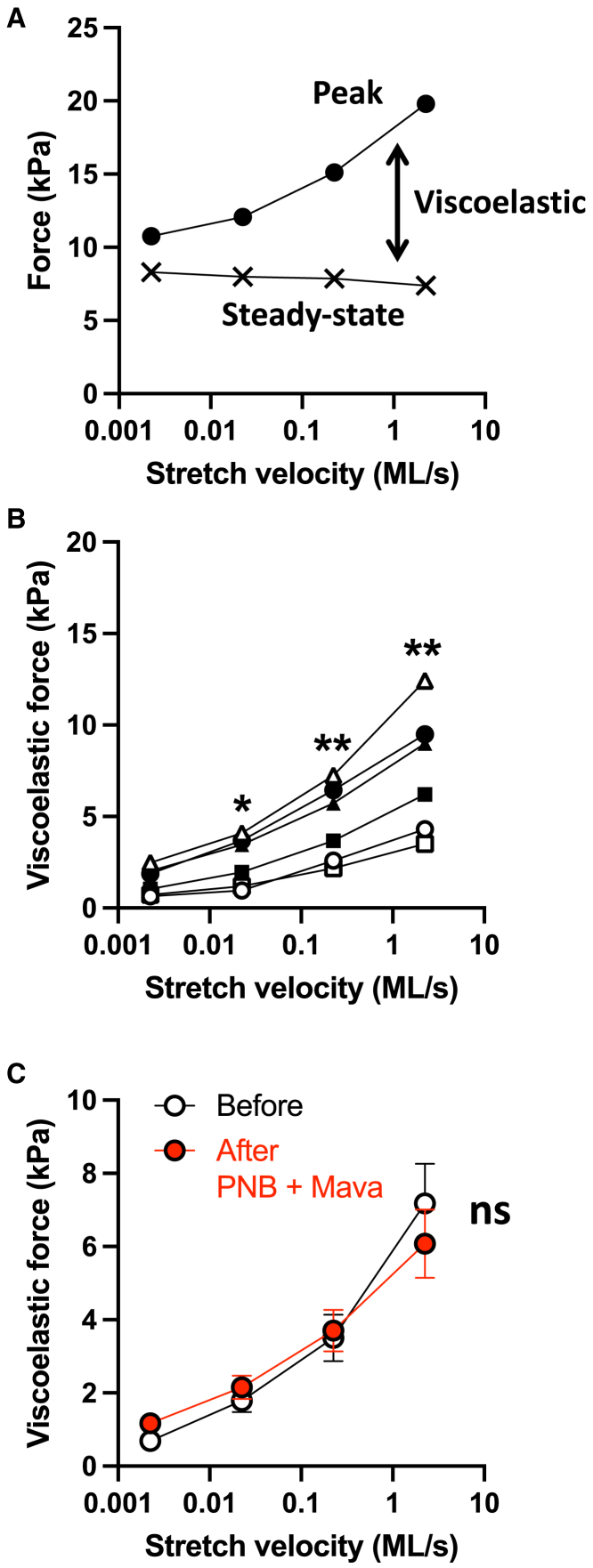
Velocity-sensitive viscoelastic property. (*A*) Example data of peak and steady-state force levels after stretches of relaxed muscle with velocities ranging over three orders of magnitude; note the logarithmic abscissa scale for stretch velocity in muscle lengths per second (ML/s). (*B*) For all muscles in relaxing conditions, the viscoelastic force (peak force minus steady-state force) increased with increasing stretch velocity (*p* < 0.01, *n* = 6, repeated-measures one-way ANOVA). Statistical comparisons are shown relative to the slowest stretch (^∗^*p* < 0.05 and ^∗∗^*p* < 0.01 with Bonferroni correction for multiple comparisons). A different symbol is used for each muscle. For relaxed muscle, the effect of stretch on viscoelastic force was not different for males (*closed symbols*, *n* = 3) versus females (*open symbols*, *n* = 3) (*p* > 0.05, repeated-measures two-way ANOVA). (*C*) Pooled data (mean ± SE, *n* = 6) comparing relaxed muscle viscoelastic force measured before versus after addition of cross-bridge inhibitors (ns, no significant difference, *p* > 0.05, repeated-measures two-way ANOVA).

The peak force and the quasi-steady-state force level 60 s later were measured after muscle stretches with velocities varying over three orders of magnitude (Fig. 2 *A*). Peak force and steady-state force were similar at the slowest stretch velocity (0.00225 ML/s, stretch duration: 100 s). However, as previously reported (12), the peak force after stretch progressively increased with increasing stretch velocity (Fig. 2 *A*). In contrast, the steady-state force level (assessed 60 s after stretch) did not appreciably change as a function of stretch velocity. Accordingly, the viscoelastic force response to stretch (peak force minus steady-state force) was observed to increase with increasing stretch velocity in all experiments (Fig. 2 *B*). The dependence of the viscoelastic force response on stretch speed was not linear.

There was not a significant male versus female difference observed in the effect of stretch velocity on viscoelastic force (Fig. 2 *B*).

The viscoelastic force observed in relaxed muscle as a function of stretch velocity was not significantly changed after the addition of the cross-bridge inhibitors PNB (50 *μ*M) plus Mava (50 *μ*M) (Fig. 2 *C*). This finding suggests that in the absence of inhibitors, cross-bridges did not contribute to the observed viscoelastic component of the force response (e.g., via residual cross-bridge attachment). Moreover, the inhibitors did not appreciably change the muscle mechanical properties. Finally, the similarity of the muscle response to stretches before versus after inhibitors indicates that the processes determining muscle viscoelasticity (e.g., uncoiling and then refolding of titin) were reversible.

### Inhibition of force development by PNB plus Mava

**Figure 3:**
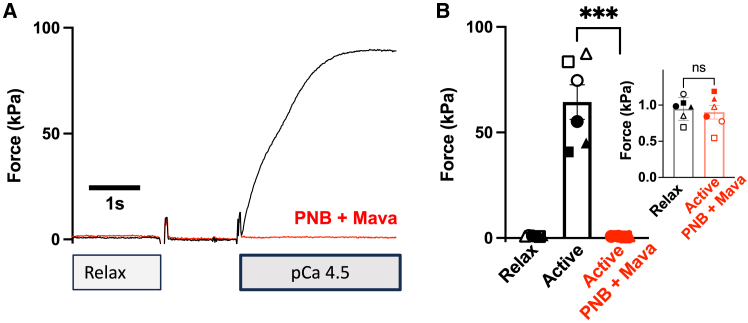
Inhibitors PNB + Mava eliminated force development. (*A*) Example records of muscle force during a transition from low Ca^2+^ solution (relax) to high Ca^2+^ solution (pCa 4.5). Note that the force record was affected by motion artifacts during the transition between solutions (≈1.5 s). In pCa 4.5 solution, the development of force in the absence of inhibitors (*black trace*) was completely prevented in the presence of the inhibitors PNB plus Mava (*red trace*). (*B*) Pooled data (mean ± SE, *n* = 6) show that muscle force at pCa 4.5 (active) was abolished by the inhibitors PNB + Mava (^∗∗∗^*p* < 0.001, paired *t*-test). A different symbol is used for each muscle. Solid symbols show males, and open symbols show females. The inset with the expanded *y* axis scale shows no difference in force level with low Ca^2+^ versus high Ca^2+^ in the presence of inhibitors (ns, *p* > 0.05, *n* = 6, paired *t*-test).

Fig. 3 *A* shows an example record of Ca^2+^-activated force development before and after incubation of trabeculae with the cross-bridge inhibitors PNB (50 *μ*M) plus Mava (50 *μ*M) (Fig. 3 *A*). Before adding the inhibitors, the maximum Ca^2+^-activated force was high; however, in the presence of the inhibitors, the force in relaxing solution was not increased after transfer to pCa 4.5 solution (Fig. 3 *A*).

For all experiments, the Ca^2+^-activated force was 64 ± 8 kPa (*n* = 6; Fig. 3 *B*). Interestingly, the Ca^2+^-activated force was higher in trabeculae from females (82 ± 4 kPa, *n* = 3) than in trabeculae from males (47 ± 4 kPa, *n* = 3, *p* = 0.004, unpaired *t*-test) (Fig. 3 *B*). However, in the presence of the inhibitors, the force level measured in relaxing solution (0.95 ± 0.06 kPa, *n* = 6) was not increased when the muscle was transferred to activating solution (0.9 ± 0.1 kPa, *n* = 6, *p* > 0.05, paired *t*-test) (Fig. 3 *B*, *inset*). Thus, the combination of inhibitors PNB plus Mava completely abolished Ca^2+^-activated force development.

### Ca^2+^ increased viscoelastic force response to stretch

**Figure 4:**
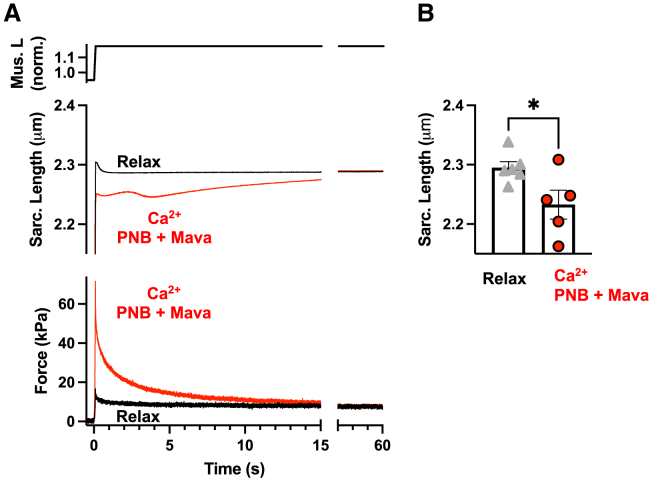
Viscoelastic property was sensitive to Ca^2+^. (*A*) Example records of muscle length, sarcomere length, and force in the presence of PNB + Mava. Peak force after a large amplitude muscle stretch was markedly increased by high Ca^2+^ level (*red trace*) compared to low Ca^2+^ level (relax). (*B*) Pooled data (mean ± SE, *n* = 5) show that sarcomere length measured immediately after stretch was not increased as much in high Ca^2+^ as in low Ca^2+^ (relax) (^∗^*p* < 0.05, paired *t*-test).

Fig. 4 *A* shows typical records of changes in sarcomere length and muscle force in response to a 20% muscle stretch at the fastest stretch velocity (2.25 ML/s, stretch duration: 0.1 s), followed by a 60 s length hold. In the presence of the cross-bridge inhibitors, records were obtained, both in low Ca^2+^ (black trace) and then in high Ca^2+^ (red trace).

In high Ca^2+^, the peak force after stretch was markedly increased compared to that with low Ca^2+^. However, after stress relaxation, the force for both high and low Ca^2+^ decreased to the same steady-state force 60 s after stretch. Thus, the high Ca^2+^ level mediated a large increase in muscle viscoelasticity. Consistent with a Ca^2+^-mediated increase in muscle viscoelasticity, muscle stretch in high Ca^2+^ did not increase the sarcomere length as much as in low Ca^2+^ (Fig. 4 *B*). However, in high Ca^2+^, sarcomere length increased during stress relaxation and, after 60 s, was not different in high Ca^2+^ (2.3 ± 0.02 *μ*m) versus low Ca^2+^ (2.27 ± 0.01 *μ*m, *n* = 5, *p* > 0.05, paired *t*-test).

**Figure 5:**
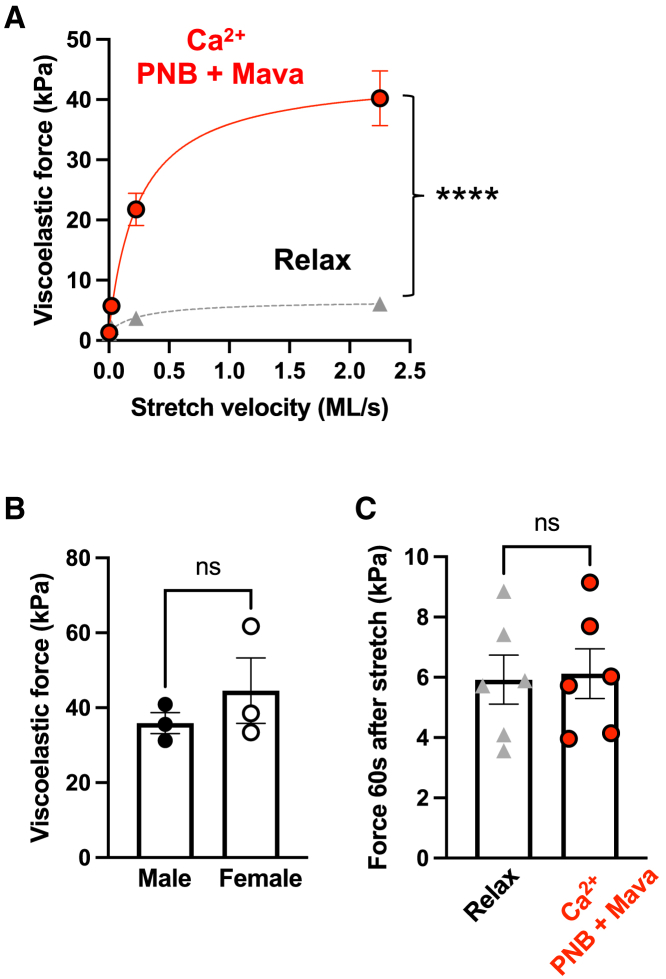
Velocity- and Ca^2+^-sensitive viscoelastic property. (*A*) Pooled data (mean ± SE, *n* = 6) showing the relation of muscle viscoelastic force (peak-minus steady-state level) versus muscle stretch velocity for experiments performed in the presence of cross-bridge inhibitors and low Ca^2+^ (relax) or high Ca^2+^ (^∗∗∗∗^*p* < 0.0001, repeated-measures two-way ANOVA). (*B*) Viscoelastic force response to the fastest stretch (velocity: 2.25 ML/s) of trabeculae from males versus females in the presence of cross-bridge inhibitors and high Ca^2+^ (ns, *p* > 0.05, *n* = 3 per group, unpaired *t*-test). (*C*) Force level 60 s after 0.1 s stretch/hold protocol in the presence of cross-bridge inhibitors at both low Ca^2+^ (relax) and high Ca^2+^ (ns, *p* > 0.05, *n* = 6, paired *t*-test).

For all experiments, the viscoelastic force response to stretch (peak force minus steady-state force 60 s after stretch) was increased with stretch velocity (Fig. 5 *A*) and was increased much more with high Ca^2+^ than with low Ca^2+^. The extrapolated maximum with high Ca^2+^ (44.4 kPa) was >sixfold higher than with low Ca^2+^ (6.6 kPa) (Fig. 5 *A*). However, the estimated stretch velocity associated with a half-maximal increase in viscoelastic force was similar in high Ca^2+^ (0.25 ± 0.02 ML/s) versus low Ca^2+^ (0.28 ± 0.04 ML/s, *n* = 6, *p* > 0.05, paired *t*-test), suggesting that the viscoelasticity mechanism was similar at both high and low Ca^2+^. The observed dependence of force development on stretch velocity is explored in a theoretical model developed by Jezek et al. (24).

Despite the higher developed force noted for trabeculae from females versus males, there was no sex difference in the viscoelastic force response to stretch (Fig. 5 *B*).

The force measured 60 s after stretch was not increased by Ca^2+^ (Fig. 5 *C*). This finding is consistent with the observed absence of force development in the presence of the inhibitors and also suggests that there is no effect of Ca^2+^ on the muscle elastic property.

### Relationship of muscle viscoelastic property to Ca^2+^ level

**Figure 6:**
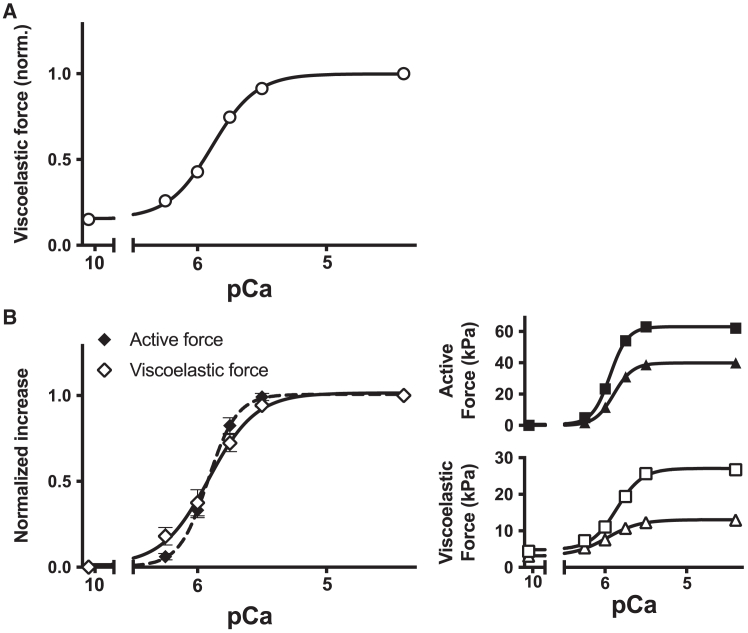
Ca^2+^ regulation of viscoelastic property. (*A*) Effect of Ca^2+^ level (expressed as pCa, −log[Ca^2+^]) on the viscoelastic force response to the fastest stretch (velocity: 2.25 ML/s) in the presence of cross-bridge inhibitors (mean ± SE, *n* = 6, error bars smaller than symbols). For each experiment, viscoelastic force was normalized to the force measured at pCa 4.5. Pooled data were fit to the Hill equation. (*B*) In two additional experiments, the developed force-pCa relation was measured in the absence of inhibitors (closed symbols). Then, in the same two muscles, the viscoelastic force-pCa relation was measured in the presence of inhibitors (*open symbols*). Values were expressed as an increase relative to the values in relaxing solution (means ± SE, *n* = 2). Muscle length was the same (1.175 Lo) for both measurements. Nonnormalized values for both experiments are shown in the graphs on the right (different symbol for each experiment).

The dependence of the viscoelastic force component on the Ca^2+^ level was assessed using the response to the fastest stretch velocity (2.25 ML/s) over a range of Ca^2+^ levels. The viscoelastic force versus pCa relation (Fig. 6) had a sigmoidal form that was well described by the Hill equation (EC_50_ = 1.3 *μ*M, *n*H = 2.5, *n* = 6). The viscoelastic force for relaxed muscle was 16% of the value for maximal Ca^2+^ activation. This relationship did not differ between males and females (*p* = 0.45, *n* = 3, two-way repeated-measures ANOVA).

In a subset of experiments (*n* = 2), the effect of Ca^2+^ on active force development (in the absence of inhibitors) was directly compared in the same muscles to the effect of Ca^2+^ on the viscoelastic force response to stretch (in the presence of the inhibitors) (Fig. 6 *B*). To minimize length-dependent effects on Ca^2+^ activation, both force development and the viscoelastic force response to stretch were measured at the same muscle length (1.175 Lo). The viscoelastic force-pCa relation was similar in form to the developed force-pCa relation, and their EC50 values were similar (1.22 ± 0.14 vs. 1.21 ± 0.06 *μ*M) (Fig. 6 *B*).

### Temperature-independent muscle viscoelastic property

**Figure 7:**
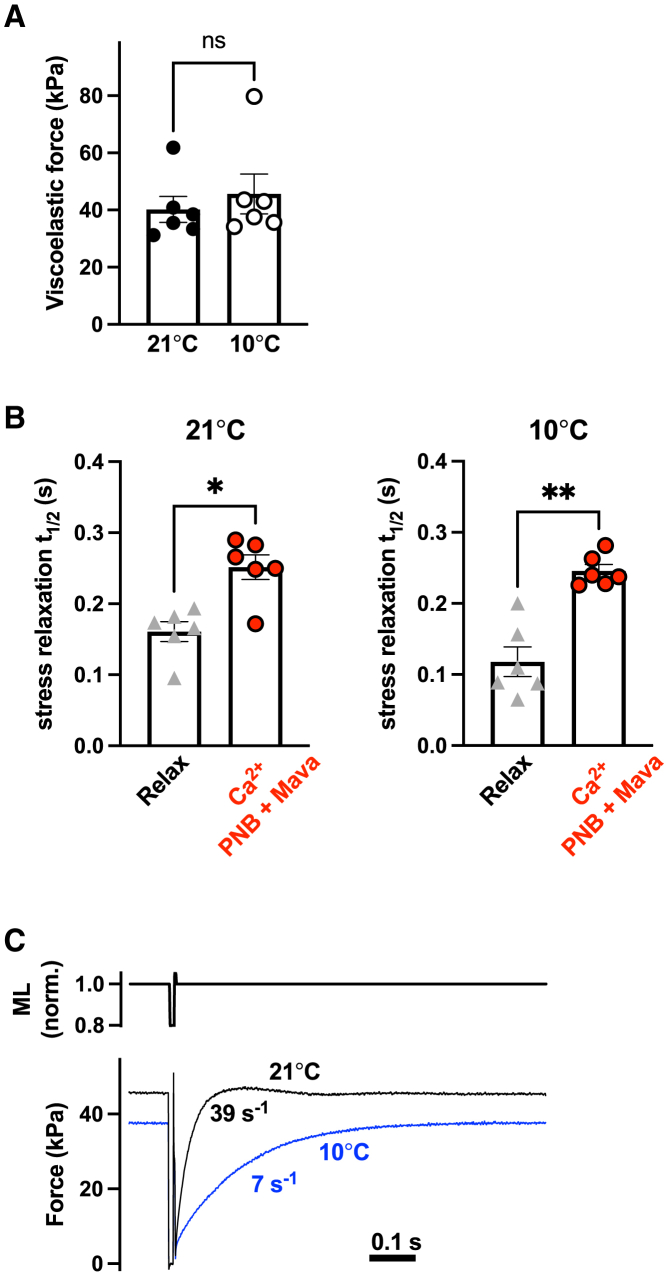
No effect of temperature on Ca^2+^-activated viscoelastic property. (*A*) Ca^2+^-activated viscoelastic force was not decreased by lowering temperature from 21°C to 10°C (ns *p* > 0.05, *n* = 6, paired *t*-test). (*B*) Stress relaxation half time (t_½_) measured directly from records for trabeculae in the presence of inhibitors and both low Ca^2+^ (relax) and high Ca^2+^ and with temperature set first to 10°C and then 21°C (^∗^*p* < 0.05 and ^∗∗^*p* < 0.01, *n* = 6, paired *t*-tests). (*C*) Low temperature reduced developed force and slowed the rate constant of force redevelopment after cross-bridges were mechanically disrupted.

As a further check for a contribution of cross-bridge activity to the increased viscoelastic force response to stretch at the high Ca^2+^ level, we assessed the effect of temperature. Reducing the temperature from 21°C to 10°C did not reduce the viscoelastic force response to stretch assessed at the fastest stretch velocity (40 ± 4.5 vs. 46 ± 7 kPa) (Fig. 7 *A*).

We also assessed the effect of temperature on the time course of stress relaxation after a stretch. For both temperatures, the half-time of stress relaxation (t_½_) with the high Ca^2+^ level was significantly higher than that with the low Ca^2+^ level (Fig. 7 *B*). However, with the high Ca^2+^ level, t_½_ was not affected by reducing the temperature from 21°C to 10°C (0.25 ± 0.18 vs. 0.24 ± 0.1 s, *n* = 6, *p* > 0.05, paired *t*-test). In contrast, cross-bridge activity is known to be impaired by low temperature (25,26). Therefore, a lack of sensitivity to temperature is consistent with the stress relaxation process not involving cross-bridge activity. To evaluate the effect of temperature on cross-bridge activity in our preparation, for one experiment where force was measured in the absence of an inhibitor, as expected, the maximum force level was lower at 10°C than at 21°C (37 vs. 46 kPa) (Fig. 7 *C*). Moreover, the rate constant for force redevelopment after mechanically disrupting cross-bridges was appreciably slower at 10°C (7 s^−1^) than at 21°C (39 s^−1^) (Fig. 7 *C*). To minimize the potential impact from deterioration of the preparation with multiple activations, we studied contraction first at 10°C and then at 21°C.

In summary, viscoelastic force was not sensitive to temperature, but actively developed force was sensitive to temperature. This difference suggests that different mechanisms are involved in the development of active force versus viscoelastic force and further suggests that cross-bridge cycling does not contribute to the Ca^2+^-sensitive viscoelastic property.

Finally, in the absence of inhibitors, the rate constant for force redevelopment after mechanically disrupting cross-bridges was 35 ± 5 s^−1^ (*n* = 6), corresponding to a t_½_ of 0.02 ± 0.005 s. Thus, the time course of force redevelopment was approximately an order of magnitude faster than the time course of stress relaxation (Fig. 7 *B*), reinforcing the idea that force development and muscle viscoelasticity involve different underlying phenomena.

### Ca^2+^ effect on muscle viscoelastic property requires intact myofilaments

**Figure 8:**
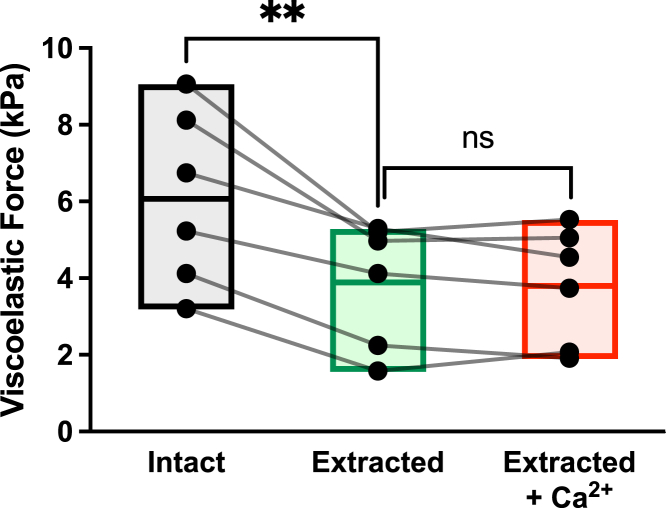
Effect of Ca^2+^ to increase viscoelastic force was lost after extraction of myofilaments. Viscoelastic force measured in the presence of inhibitors at low and high Ca^2+^ levels for trabeculae before (intact) and after high-salt extraction of the myofilaments (*shaded boxes* indicate mean and high and low values, ^∗∗^*p* < 0.01 and ns *p* > 0.05, *n* = 6, paired *t*-tests).

For trabeculae subjected to a high-salt extraction protocol designed to remove the myofilaments, the viscoelastic force measured after the most rapid stretch was reduced from 6.1 ± 0.9 to 3.9 ± 0.7 kPa (*n* = 6; Fig. 8). This suggests that at the extended muscle length attained after a large 20% stretch, the viscoelastic force measured after the stretch is partly attributed to myofilament-based structures and partly to nonmyofilament structures, such as the extracellular matrix. The relative contributions of forces associated with myofilament versus nonmyofilament structures are addressed in the theoretical study by Jezek et al. (24).

Importantly, after myofilament extraction, for high Ca^2+^ versus low Ca^2+^, there was no difference in the viscoelastic force response to stretch (Fig. 8). This suggests that the effect of Ca^2+^ to increase the viscoelastic response to muscle stretch involves a myofilament property.

## Discussion

The major finding of this study is that Ca^2+^ regulates a viscoelastic property of cardiac muscle independent of the effect of Ca^2+^ on cross-bridge cycling. This phenomenon considerably expands the role of Ca^2+^ in impacting cardiac muscle mechanical properties, where Ca^2+^ mediates both activation of contraction and an increase in muscle stiffness. This effect of Ca^2+^ on a muscle’s viscoelastic property might influence the mechanical response of myocardium to fluctuating Ca^2+^ levels during activation. Since the Ca^2+^-mediated effect on the viscoelastic property of cardiac muscle was significant only at Ca^2+^ levels that are greater than diastolic [Ca^2+^], the degree to which the Ca^2+^-mediated response is relevant in diastole depends on how rapidly the Ca^2+^-mediated effect is reversed. Building on our observations, Jezek et al. have developed a theoretical model to explore the potential effects of Ca^2+^ on the passive mechanical properties of the myocardium (24).

### Viscoelastic property of relaxed muscle

We observed the familiar biphasic stress response to a ramp increase in muscle length. Force increased during the stretch and reached a peak at the completion of the stretch. After the ramp stretch, when the muscle length was held at a constant final length, the stress decayed to a steady level. Stress relaxation of cardiac muscle (12,13,14,15) is consistent with a viscoelastic material response.

The observed peak stress increased with increasing stretch velocity, even as the final length was the same for all ramp speeds. This dependence of peak stress on the stretch velocity was nonlinear (Fig. 2 *B*). Importantly, the magnitude of the viscoelastic response was observed to be sensitive to stretch velocity over a physiologically relevant range of stretch velocities and may be relevant to the mechanical function of the beating heart.

Multiple structural elements contribute to cardiac muscle viscoelastic properties, with approximately equal contributions reported to arise from titin, actin, microtubules, and intermediate filaments (8). For muscles subjected to high strains, such as the stretch protocol used in this study, a significant contribution also arises from the extracellular matrix (8).

A theoretical analysis of the observed stress response to stretch suggests that the passive stress relaxation proceeds over a power-law time course that is governed in part by the molecular mechanics of the elastic domain of titin in the I band of the sarcomere (24).

### Inhibition of cross-bridge cycling

Characterizing the effect of Ca^2+^ on the muscle viscoelastic property relies crucially on effectively inhibiting Ca^2+^ activation of cross-bridge cycling. Preliminary studies revealed that 50 *μ*M PNB alone did not completely inhibit Ca^2+^-activated force development (the residual force was ∼10%). Therefore, to completely inhibit force development, the current study combined two inhibitors: 50 *μ*M PNB plus 50 *μ*M Mava.

Effective inhibition of contraction was achieved by the combination of PNB plus Mava. Specifically, Ca^2+^-mediated force was abolished in the presence of the inhibitors and a high Ca^2+^ level. Furthermore, the force level 60 s after stretch was not increased relative to relaxed muscle, indicating no active force generation.

### Ca^2+^ activation of cardiac muscle viscoelastic property

Previous studies have suggested that an interaction between the PEVK domain of titin and actin modulates the passive tension of both cardiac and skeletal muscle (1,2,3). The titin-actin interaction was reported to be sensitive to the level of muscle activation by Ca^2+^ (1,2,4,5,6). Moreover, the rigidity of the PEVK domain was increased by Ca^2+^ (3). Together, these reports suggest that muscle passive mechanical properties may be modulated by Ca^2+^.

Consistent with these previous reports, recent studies found that electrical stimulation of frog skeletal muscle increased the passive component of stiffness after active force development was prevented by the cross-bridge inhibitor PNB (7). An increase in the intracellular Ca^2+^ level after electrical stimulation was suggested to increase muscle stiffness mediated by switching of titin from an extensible spring (OFF state) to a mechanical rectifier (ON state) that allows shortening but has an elevated resistance to stretch (7). The titin ON state and increased muscle stiffness were hypothesized to involve cross-linking of flexible titin to relatively stiffer actin filaments (7).

The current study directly tested the effect of activator Ca^2+^ levels on cardiac muscle passive stiffness. In the presence of the cross-bridge inhibitors PNB plus Mava, a high Ca^2+^ level increased the magnitude of the viscoelastic force response to muscle stretch by ≈sixfold relative to the relaxed state. The high viscoelastic force was measured immediately after the stretch but dissipated after 60 s.

Several lines of evidence suggest that the Ca^2+^-mediated increase in the muscle viscoelastic property did not involve effects due to cross-bridge cycling: 1) force development was completely abolished, 2) low temperature impaired cross-bridge cycling but did not reduce the viscoelastic force or slow the time course of viscoelastic force relaxation, and 3) the time course of viscoelastic force relaxation was an order of magnitude slower than expected for a cross-bridge-mediated effect.

The data from the current study were analyzed using a computational model that simulates stress relaxation based on the unfolding of serial globular elements in the titin chain, where each unfolding event introduced slack, reduced stress, and reduced the rate of subsequent unfolding (24). The model includes Ca^2+^-mediated attachment of the PEVK domain of titin to the actin filament and a Ca^2+^-mediated stabilization of folded conformations of serial domains in the titin chain, capturing both the observed dependence of peak stress on Ca^2+^ level and the kinetics of stress relaxation (24).

Using a similar mechanical protocol to the current study, previous studies tested cardiac muscle preparations in which the actin filaments had been depolymerized using gelsolin (4,5). Interestingly, in the absence of actin filaments, the cardiac muscle response to stretch did not manifest a large increase in viscoelasticity with elevated Ca^2+^ levels (4,5). Thus, the large Ca^2+^-mediated increase in viscoelasticity we observed likely requires the presence of actin filaments.

The effect of Ca^2+^ to increase the muscle viscoelastic property was abolished after a high-salt protocol to extract the myofilaments, suggesting that the Ca^2+^ effect involves a myofilament-based mechanism.

The dependence of viscoelastic stiffness on the level of activator Ca^2+^ followed a sigmoidal relationship with the same sensitivity to Ca^2+^ as observed for Ca^2+^ activation of myofilament force development. This suggests a common regulatory mechanism resulting in a coordinated effect of Ca^2+^ to both increase force development and increase viscoelastic stiffness.

Our finding of no effect of Ca^2+^ on the muscle elastic property (evidenced by no effect on force measured 60 s after stretch) is consistent with a previous study of rat myocardium (4). However, the effect of Ca^2+^ on muscle elasticity was reported to be titin-isoform dependent (4), with no effect in rat myocardium (expressing N2B titin) but increased elasticity in bovine left atrium (N2BA) and skeletal muscle (N2A) (2,4).

### Sex difference

Trabeculae from both males and females were tested. Muscle viscoelastic and elastic properties were not different in males versus females. Interestingly, trabeculae from females developed significantly higher forces than those from males. The reason for this difference is not clear. Trabeculae from females tended to be thinner, which might result in some overestimation of force development. Further studies of this sex difference in force development are warranted.

### Limitations of the study

We did not directly test the role of actin-titin association in the Ca^2+^-mediated increase in viscoelasticity, for example, by measurements before versus after depolymerizing actin filaments with gelsolin.

Mava stabilizes an autoinhibited structural state of myosin termed the interacting heads motif, where the myosin heads are folded back onto the filament backbone (27,28). This structural effect of Mava might affect the mechanical properties of the thick filament. Whether such an effect of Mava could contribute to the Ca^2+^-mediated increase in viscoelasticity was not determined in this study. Similarly, although PNB + Mava abolished active force development, we also cannot exclude the possibility that cross-bridges accumulate in weak binding states that contribute to the Ca^2+^-mediated increase in viscoelasticity.

The loss of calcium-dependent viscoelasticity due to high-salt extraction suggests that a myofilament property is involved. However, we cannot exclude that other factors (such as extracellular matrix proteoglycans) might be lost due to high-salt extraction.

This study found that the Ca^2+^-mediated increase in viscoelasticity was not sensitive to temperature in the range of 10°C–21°C. Although we confirmed an expected effect of temperature on contraction force and kinetics in one experiment, we did not perform a full survey of the effect of temperature on contraction.

This study did not determine the effect of sarcomere length on the viscoelastic response to stretch or determine if other ions, besides Ca^2+^, mediate an increase in viscoelasticity.

Finally, it is unclear how the viscoelastic modulus we observed for activated muscle in the presence of inhibitors would compare to activated muscle in the absence of inhibitors.

## Conclusion

In addition to Ca^2+^ activation of contraction, Ca^2+^ also markedly increased the apparent viscoelastic force response to rapid stretch of cardiac muscle. This behavior opens a new window on Ca^2+^ modulation of cardiac muscle mechanical properties that may have implications for understanding both systolic and diastolic properties.

## Acknowledgments

This work was supported by 10.13039/100000738Department of Veterans Affairs Merit Review Award I01BX000740 (A.J.B.) and 10.13039/100000050National Heart, Lung, and Blood Institute grant R01 HL154624 (A.J.B., D.A.B., and N.C.C.).

## Author contributions

Designed research, A.J.B., F.J., N.C.C., P.C.S., and D.A.B.; performed research, A.J.B. and O.Y.L.; analyzed data, A.J.B., F.J., and D.A.B.; wrote the paper, A.J.B., F.J., P.C.S., N.C.C., and D.A.B.

## Declaration of interests

The authors declare no competing interests.
